# Ancient genomics study reveals low HLA diversity in eastern hunter-gatherers

**DOI:** 10.1186/s13059-026-04214-8

**Published:** 2026-07-29

**Authors:** Onur Özer, Nicolas Antonio da Silva, Magdalena Haller-Caskie, Daniel Anton Myburgh, Henny Piezonka, Anastasia Khramtsova, Elena L. Kostyleva, Maria V. Dobrovol’skaya, Sergei V. Vasilyev, Elizaveta Veselovskaya, John Meadows, Almut Nebel, Ben Krause-Kyora

**Affiliations:** 1 Institute of Clinical Molecular Biology, Kiel University, Kiel, 24105 Germany; 2 Institute of Prehistoric Archaeology, Free University of Berlin, Berlin, 14195 Germany; 3 Institute of Pre- and Protohistoric Archaeology, Kiel University, Kiel, 24118 Germany; 4 Ivanovo State University, Ivanovo, 153025 Russia; 5 Institute of Archaeology of the Russian Academy of Sciences, Moscow, 117292 Russia; 6 Institute of Ethnology and Anthropology of the Russian Academy of Sciences, Center of Physical Anthropology, Moscow, 119334 Russia; 7 Leibniz-Laboratory for AMS Dating and Stable Isotope Research, Kiel University, Kiel, 24118 Germany; 8 Leibniz-Zentrum für Archäologie, Schloss Gottorf, Schleswig, 24837 Germany

**Keywords:** Ancient DNA, Eastern hunter-gatherers, HLA diversity, Immunogenetics

## Abstract

**Background:**

Eastern hunter-gatherers (EHG) contributed varying proportions of genetic ancestry to many modern Europeans. However, how this contribution shaped the human leukocyte antigen (HLA) gene diversity remains unexplored. Sakhtysh in western Russia comprises one of the largest EHG-affiliated burial complexes. The site's continuous use offers a rare opportunity to investigate the genetic history, immune-gene diversity, and pathogen exposure within a hunter-gatherer population.

**Results:**

Genome-wide data from 36 individuals (spanning Lyalovo–Volosovo traditions, 6800–4800 cal BP) place the Sakhtysh population within the EHG cluster, with high genetic affinity to contemporaneous groups across northwestern Russia and the eastern Baltic. This genetic composition remained stable for over two millennia and there is no evidence of admixture with neighboring western hunter-gatherer or farmer groups. We report HLA genotypes for 27 Sakhtysh individuals, representing the first true HLA calls from ancient hunter-gatherers. HLA diversity is low in the Sakhtysh population, likely due to their small population size. Specific alleles like B*27:05 are observed at high frequencies (~ 50%), despite being relatively rare in modern populations, suggesting shifts in selective pressures. Pathogen screening identified a restricted spectrum of causal agents including possible zoonoses with *Erysipelothrix rhusiopathiae* and chronic infections with hepatitis B virus and parvovirus B19.

**Conclusions:**

The Sakhtysh community shows genetic continuity across cultural transitions. This population exhibits limited HLA diversity and unexpectedly high frequency of certain alleles. These patterns were likely influenced by low pathogen diversity and possible adaptation to specific environmental factors.

**Supplementary Information:**

The online version contains supplementary material available at 10.1186/s13059-026-04214-8.

## Background

Eastern hunter-gatherers (EHG) were a culturally and genetically distinct group in Europe, from approximately 14000 cal BP to 5000 cal BP [1]. They inhabited an area whose largest extent stretched from the Baltic Sea and the Black Sea in the west to the Ural Mountains, including the Pontic-Caspian Steppe in the south [2, 3]. Their subsistence strategies, such as fishing, hunting and gathering, reflect their ability to thrive in different environments, ranging from coastal areas to forests and grasslands. The EHG genomic composition consists mainly of Ancient North Eurasian (ANE) ancestry introduced from Siberia and a smaller Paleolithic component from a source related to the Villabruna cluster [1, 4, 5]. As the Villabruna/Oberkassel cluster gave rise to the formation of the western hunter-gatherers (WHG), it reflects the common ancestry of both the EHG and WHG, which can be dated to around 14000 cal BP [5].

The EHG interacted with the WHG around the eastern Baltic and the early farmers (EF) around the lower Dnipro Valley, but remained largely unadmixed [6, 7]. From 5000 cal BP onwards, the situation changed with the expansion of the Steppe pastoralists, which ultimately resulted in the disappearance of the EHG as an independent cultural group [2, 8, 9]. About half of the Steppe ancestry is derived from the EHG. As Steppe pastoralists interbred with local farmers in central Europe, the genomic signature of the EHG is still visible today [2]. A hallmark of the EHG contribution to the modern European gene pool is the Y-chromosome haplogroup R1b1a1, which is the most frequent paternal lineage in men across the continent today [10].

The archeological site of Sakhtysh in the Upper Volga region of western Russia is of particular interest for a genomics study of EHG. Sakhtysh burials are associated with several cemeteries and collective graves, the most extensive one being Sakhtysh IIa. They were archeologically attributed to the Lyalovo and Volosovo cultural traditions, spanning from 6800 to 4800 cal BP [11]. The Lyalovo culture is characterized by its pit-comb pottery, which was present only in its earliest phase and pit graves that included a limited number of bone tools and tooth ornaments as grave goods [12]. The later Volosovo culture represents a ceramic tradition largely unrelated to Lyalovo, with distinct decorative motifs and the introduction of metal-based tools, especially towards the late phases [13]. Sakhtysh is renowned as one of the most notable agglomerations of burials for hunter-gatherer-fisher groups in north-eastern Europe [14, 15], allowing us to examine multiple individuals from a large communal burial complex. This approach offers the potential to substantiate the genetic heritage of the EHG on modern populations, which is still limited by ancient DNA (aDNA) datasets that have been generated from geographically dispersed individuals [1–3].

Here, we analyzed genomic data from 36 individuals from Sakhtysh, examining the genetic affinities of individuals over time (Lyalovo *vs.* Volosovo traditions) within the site and with neighboring populations. In addition, we performed kinship analysis and screened the samples for pathogens. Finally, we focused on highly polymorphic human leukocyte antigen (HLA) genes that are central mediators of adaptive immune responses. Variation in HLA genes is associated with many autoimmune and infectious diseases and is presumed to evolve in response to pathogen pressures [16]. Despite the relatively well-characterized HLA diversity in modern populations, the lack of temporal data hinders the investigation of HLA evolution. HLA genotypes from a few Neolithic farming groups exhibit considerable differences from each other and from modern Europeans [17–19]. So far, no such genotype information exists for hunter-gatherers except for a few alleles inferred by tag-SNP approaches [20, 21]. While tag-SNP-based inference enables frequency estimation in low-coverage genomes, it is limited by incomplete marker availability and variable specificity between different populations. Using a targeted enrichment procedure, we generated genuine HLA genotypes (*n* = 27), allowing direct comparisons with other populations and providing insights into the possible effects of admixture and/or selection on the ancient HLA allele pool.

## Results

### Genetic continuity and lack of high-degree kinship in Sakhtysh

For 43 individuals, the generated shotgun sequencing data were mapped to the human genome build hg19. Pseudohaploid genotypes for SNPs of the 1240K and Human Origins (HO) panels were generated. Only samples with more than 20000 genotyped SNPs from the 1240K panel were included in the population genetic analyses (32 out of 43; Additional file 1: Table S1). We added published data from four additional Sakhtysh individuals [1] (Additional file 1: Table S1), resulting in a total number of 36. These samples were grouped according to chronological classifications defined in [11]: Lyalovo (*n* = 5), Early Volosovo (*n* = 10), Volosovo (*n* = 2), Late Volosovo (*n* = 16) and Volosovo-transitional (*n* = 3). Two individuals from the Volosovo group could belong to either earlier or later periods and were therefore kept separately.

**Fig. 1:**
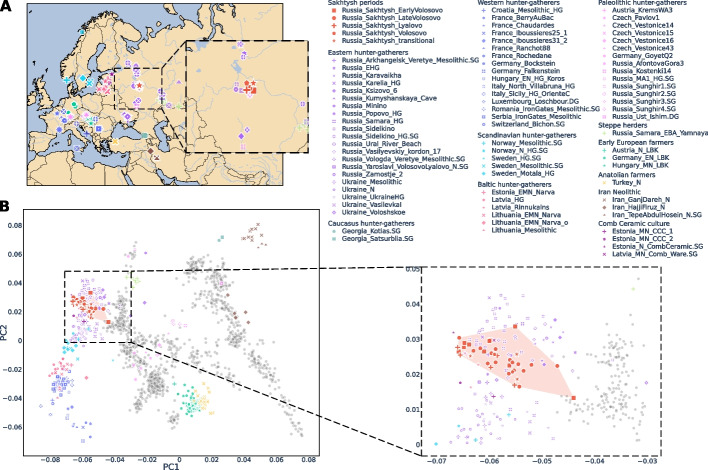
**A** Geographic location of Sakhtysh and chosen published ancient individuals from west of the Ural Mountains to central Europe. **B** Principal component analysis (PCA) of present-day West Eurasian populations from the Human Origins dataset (in light grey) and projection of selected ancient individuals

A principal component analysis (PCA) where ancient individuals were projected onto the modern West Eurasian genetic variation placed the Sakhtysh individuals from all phases within a cloud of published Russian EHG samples from Sidelkino, Vasilyevskiy kordon 17, Popovo and Karelia Yuzhnyy Oleni Ostrov (Fig. 1). Outgroup f_3_ statistics further revealed a high similarity between Sakhtysh and EHG groups located along the Baltic Sea, particularly those affiliated with the Comb Ceramic Culture (Additional file 2: Fig. S1). Ancestry decomposition using unsupervised admixture showed that the Sakhtysh individuals displayed two ancestral signals, one maximized in the ANE and the other in the WHG (Additional file 2: Fig. S2). f_4_ statistics uncovered that Sakhtysh exhibited a relatively higher WHG affinity (Additional file 2: Fig. S3) and concomitantly lower ANE affinity (Additional file 2: Fig. S4) than other EHG groups (e.g., Russia_Samara_HG, Russia_Popovo_HG and Russia_EHG). Feasible qpADM models with Sidelkino as EHG proxy showed 14–18% WHG ancestry for Sakhtysh (Additional file 1: Table S2). qpAdm models using ANE and WHG proxies as sources for Sakhtysh suggested up to 38% WHG ancestry for Sakhtysh (Additional file 1: Table S2), a proportion that resembles values estimated by the unsupervised admixture method (Additional file 2: Fig. S2). However, these models were unfeasible (*p*-value < 0.05). This has been previously noted when modeling other EHG groups and indicates that the available proxies do not adequately represent the specific ancestries that contributed to the EHG/Sidelkino cluster formation [5].

To assess the genetic continuity among individuals from the different phases in Sakhtysh, we performed an f_3_-outgroup test in the format f_3_(IIa grave 40, X; Mbuti), where “IIa grave 40” is the second best covered and the second oldest individual in the dataset and X is the test individual. This test revealed little variation in the f_3_ score between the oldest and youngest individuals from Sakhtysh (Additional file 2: Fig. S5). We further validated this pattern using pairwise f_4_ statistics in the format f_4_(Mbuti, sampleA; sampleB, outgroup) across multiple outgroups. The resulting f_4_ values clustered tightly with no strong correlation with temporal distance between sample pairs (Additional file 2: Fig. S6). Consistent with this, no temporal structure was observed in the PCA components (Additional file 2: Fig. S7). Together, these findings indicate genetic continuity in the Sakhtysh population lasting at least two millennia.

Among the 36 individuals from Sakhtysh, no first-degree kinship was observed using READv2. Instead, our analysis revealed three second-degree related pairs, all associated with the Late Volosovo phase (Additional file 2: Fig. S8; Additional file 1: Table S3).

### Pathogen screening

We screened the metagenomic sequencing reads of all our Sakhtysh individuals (*n* = 43; Additional file 1: Table S1) for the presence of bacterial or viral DNA. In two tooth samples (taken from IIa grave 65 and IIa grave 58), more than 1000 reads were assigned to *Erysipelothrix rhusiopathiae* (Additional file 1: Table S4)*.* Mapping carried out using the whole genome reference of *E. rhusiopathiae* strain Fujisawa (NC_015601.1) revealed 1 × genome coverages of 6.0% and 19.5%, respectively (Additional file 1: Tables S4-S5) and deamination patterns distinctive of aDNA (Additional file 2: Fig. S9). Furthermore, human parvovirus B19 (B19V) and hepatitis B virus (HBV) infections were detected in four and five samples, respectively (Additional file 1: Table S1). One sample (IIa grave 42) even showed a co-infection of both HBV and B19V. Through competitive mapping against a set of five ancient references, the HBV genome in this sample was most closely associated with the "Western-Eurasian Neolithic–to–Bronze Age" (WENBA) lineage (Additional file 1: Table S6) [22]. However, due to inadequate genome coverage in the sample, its viral lineage could not be refined within a phylogenetic context. Additionally, reads assigned to nine oral bacterial species were detected in various samples potentially implicated in the formation of dental plaque and periodontitis (Additional file 1: Table S4).

### HLA diversity

Among the 43 individuals sampled, 31 had petrous bone available. In-solution DNA enrichment of the HLA region was performed for these 31 samples, 27 of which yielded sufficient data for genotyping. HLA alleles were identified for the class-I (HLA-A, -B, -C) and class-II (HLA-DPB1, -DQB1, -DRB1) loci using the TARGT pipeline [23]. HLA class-I allele calls were further confirmed with OptiType [24]. Of the 27 individuals, 13 were successfully genotyped at all six loci, while the rest had varying coverage, usually with missing HLA-DPB1 genotypes (Additional file 1: Table S1). Because all individuals exhibited similar EHG ancestry that remained stable across different periods (Additional file 2: Figs. S2 and S5), we pooled them for the downstream HLA analysis. We then evaluated whether the allele frequencies in our Sakhtysh sample conformed to Hardy–Weinberg equilibrium (HWE) at the two‑field resolution. All loci were in HWE, except for HLA‑A, which showed a statistically significant deviation (Additional file 1: Table S7).

We investigated the HLA genotypes of the Sakhtysh population in comparison to those of two Neolithic groups, namely EF and late farmers (LF) [18], as well as a modern dataset consisting of five European populations from the 1000 Genomes Project [25] and one Russian population (“Russia Nizhny Novgorod, Russians”, labeled as MR) from the Allele Frequency Net Database [26] (Additional file 1: Table S8). The HLA diversity within populations was measured using Shannon’s diversity index, both at the first-field and the second-field resolution. Compared to modern populations, the Sakhtysh individuals had the lowest diversity at each locus, even lower than that observed in the EF and LF. The only exceptions were HLA-DQB1 at second-field resolution where EF is lower than Sakhtysh and HLA-A at first-field resolution where LF is lower (Additional file 2: Figs. S10-S11). Overall, patterns of diversity across populations were similar for both levels of resolution, especially for class-I loci. This result suggests that for each first-field allelic lineage, usually one allele was common in the populations. Notable exceptions were the pairs of HLA-B*44:02/HLA-B*44:03, HLA-C*03:03/HLA-C*03:04, HLA-C*07:01/HLA-C*07:02 that were present at similar frequencies in modern Europeans (Additional file 2: Figs. S12-S13). This pattern changed for HLA class-II, especially for the HLA-DQB1 locus, for which multiple second-field alleles within a first-field allelic lineage were often observed in modern populations. As a result, the Shannon index values calculated based on first-field resolution were lower than the values for second-field resolution (Additional file 2: Figs. S10-S11).

**Fig. 2:**
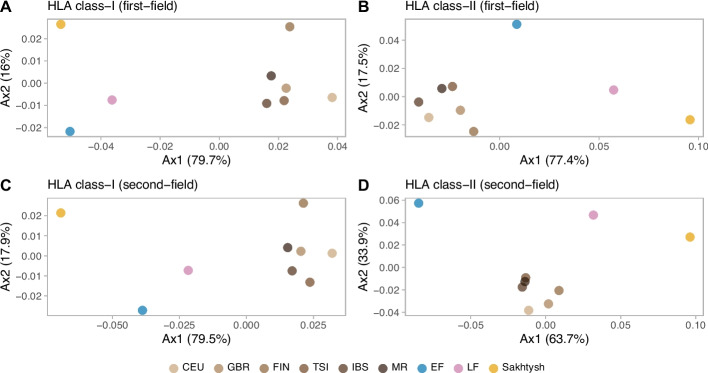
Principal coordinate plots based on pairwise F_ST_ values. HLA class-I genes used in the analysis (panels **A** and **C**) were HLA-A, HLA-B, and HLA-C, whereas HLA class-II genes (panels **B** and **D**) were HLA-DRB1 and HLA-DQB1. F_ST_ values were calculated based on both first-field (panels A and B) and second-field (panels C and D) HLA genotyping resolution. Axis labels indicate the percentage of variance explained by each axis

**Fig. 3:**
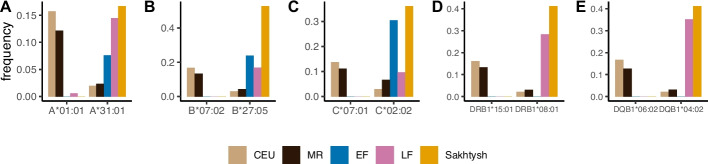
Selected HLA alleles with large frequency differences between Sakhtysh and modern Russians as well as other modern and Neolithic central European populations. Left side of each panel shows an allele common in modern, yet rare or absent in ancient populations, while the right side shows an allele with the opposite pattern. **A** HLA-A locus, **B** HLA-B locus, **C** HLA-C locus, **D** HLA-DRB1 locus, **E** HLA-DQB1 locus. CEU: Utah residents (CEPH) with Northern and Western European ancestry, MR: modern Russians from Nizhny Novgorod, LF: late farmers, EF: early farmers

Differentiation between populations was investigated using principal coordinate analysis on pairwise F_ST_ values. For class-I genes, the Sakhtysh sample exhibited the smallest F_ST_ when compared with the EF and LF (Fig. 2). This result was likely driven by alleles such as A*31:01, B*27:05, C*01:01, and C*02:02 that were very common in both Sakhtysh and the Neolithic populations relative to modern Europeans (Fig. 3, Additional file 2: Fig. S13). However, many alleles that are frequent in modern Russians or other European populations were missing in the Sakhtysh sample. Some of these alleles such as A*01:01, B*07:02, C*07:01 were not observed in the EF or LF either, highlighting the dynamic changes in the HLA allele pool in the last 7000 years. Similar to the class-I loci, large differences in class-II allele frequencies were also visible between Sakhtysh individuals and modern Europeans (Additional file 2: Fig. S12-S13). Ewens-Watterson (EW) test of neutrality suggested that different HLA loci were influenced by distinct selective pressures (Additional file 1: Table S7). HLA‑A appeared consistent with neutrality, whereas HLA‑B showed a weak trend toward directional selection. HLA‑C, DRB1, and DQB1 displayed patterns indicative of balancing selection. None of the signals of selection reached statistical significance, possibly due to the limited sample size.

Finally, we tested the effect of pooling on our HLA analysis. We removed all individuals assigned to the Lyalovo period (*n* = 4 with HLA genotypes), decreasing the time span between the youngest and oldest individual to ~ 800 years. Principal coordinate analysis based on pairwise F_ST_ values with the reduced dataset did not differ from the first analysis, which included all individuals (Additional file 2: Fig. S14). We further tested differences in HLA allele frequencies within the Volosovo period. F_ST_ between Early and Late Volosovo was zero. In order to investigate differences in allele frequencies between these two groups, we applied Fisher’s exact test. We ran the test for each allele without multiple testing correction and none of the alleles showed a significant difference. Overall, this analysis indicates that our results were not skewed by pooling individuals across a large time span.

## Discussion

Here, we present the paleogenomic analysis of 36 individuals from the Sakhtysh burial sites in modern-day northwestern Russia, belonging to the Lyalovo and Volosovo cultures (6800–4800 cal BP) [11]. Our results show that all Sakhtysh individuals are part of the EHG cluster (Fig. 1) with a sizeable portion of WHG-related ancestry (Additional file 2: Figs. S2-S3; Additional file 1: Table S2). This component is likely a remnant of the common Paleolithic population ancestral to both the WHG and EHG. Consistent with this interpretation, we detect no evidence of ongoing WHG introgression into Sakhtysh (Additional file 2: Fig. S3). Although the EHG came into contact with the WHG at the Baltic Sea, admixture between these groups appears to be limited and restricted to this region [7, 8]. Had any gene flow from the WHG reached Sakhtysh, it must have predated the Lyalovo period as the proportion of WHG-related ancestry does not increase across the phases (Additional file 2: Figs. S2-S4). This shared Paleolithic ancestry is also reflected in the presence of uniparental lineages typical of WHG, such as Y-chromosome haplogroup I and mitochondrial haplogroup U5 [5]. Taken together, our findings show that the Sakhtysh population falls into the broad genetic EHG cluster with little variation across the cultural transition spanning 2000 years of this burial site (Additional file 2: Figs. S5-S7).

Populations with EHG ancestry occupied a large area dominated by grassland steppe and forest, ranging from the Baltic and Black Seas to the Ural Mountains from 14000 cal BP onwards [1, 5]. Notably, Sakhtysh exhibits the highest levels of allele sharing with Lyalovo/Volosovo individuals from other Russian sites (Yaroslavl, Vasilyevsky kordon 17) as well as with EHG individuals associated with the Comb Ceramic Culture in the Baltic countries (Additional file 2: Fig. S1). In line with this observation, materials recovered at Sakhtysh, such as amber jewelry from the eastern Baltic or serpentine ornaments from the Ural mountains reflect high connectivity, including both material and genetic exchange within the region [27]. The EHG utilized sites in the Sakhtysh area for two millennia to bury their dead in formal cemeteries, continuing a practice that took hold across western, north-central and eastern Europe from ~ 9000 BP onwards (e.g., Groß Fredenwalde, Zvejnieki, Olenii Ostrov) [28]. Despite this long usage, no first-degree kinship is observed in Sakhtysh and only a few instances of second-degree relations are identified (Additional file 2: Fig. S8), all among individuals from Sakhtysh IIa categorized under the Late Volosovo period. The lack of a high degree of relatedness among individuals suggests that the Sakhtysh burial sites were used selectively and other forms of burial were also practiced that are less visible archeologically. Furthermore, the long time span the burial grounds were used decreases the possibility of finding highly related individuals.

In one adult female belonging to the Lyalovo culture and in one adult male from the Volosovo culture, we detect *E. rhusiopathiae.* This is a pathogen whose infections usually manifest as a mild skin disease (known as erysipeloid). The more severe septicemic form of the infection, often leading to endocarditis, is rarer [29]. Since the pathogen reads are generated from teeth, it can be assumed that the bacterium was present in the bloodstream, thus resulting in sepsis. Interhuman transmission of the disease has not been reported and human infections typically present as isolated cases of zoonoses, due to occupational exposure to the bacterium [30, 31]. Hunter-gatherers may have had close contact with several known *E. rhusiopathiae* hosts, including wild birds, freshwater fish, dogs, rodents, ticks, and mites [32]. Consequently, skin wounds acquired during activities such as hunting or fishing might have increased their susceptibility to infection [31]. As the positive cases originate in different periods (Lyalovo and Volosovo), this may indicate that the bacterium persisted in animal or environmental reservoirs surrounding Sakhtysh, thus allowing subsequent human re-infections to occur. In addition to the two infections with *E. rhusiopathiae*, genetic traces of both B19V and HBV are observed in four and five individuals, respectively. One individual exhibits evidence of a simultaneous infection with both viruses. The reconstruction of the HBV genome indicates its closest affiliation with the WENBA lineage, which is commonly associated with early European farmers [21]. HBV genomes belonging to this lineage have been identified in two hunter-gatherers (dated to 7200 BP and 6400 BP) from western Russia. Therefore, the detection of this lineage in Sakhtysh supports the hypothesis that the spread of WENBA outpaced the expansion of early farmers into Europe [22].

Changing environments and/or new zoonoses may have driven the adaptive evolution of prehistoric Europeans, particularly via HLA genes. The presentation of pathogenic peptides on the cell surface by HLA molecules initiates adaptive immune responses. The most distinctive feature of HLA genes is their extreme polymorphism, which is concentrated in the exons coding for those parts of the molecule that interact with the presented peptide [33]. Therefore, this high polymorphism is hypothesized to have evolved under dynamic pathogen pressures [16]. Surprisingly, the HLA diversity in the Sakhtysh sample is much lower compared to modern Europeans, even after controlling for different sample sizes (Additional file 2: Figs. S10-S11). This reduced diversity could be due to two non-mutually exclusive reasons. First, the small effective population size of the hunter-gatherers may have led to a less diverse HLA allele pool [34]. A similar pattern has also been observed in several modern populations with small effective population sizes such as Taiwanese aborigines or Amerindians, suggesting a strong effect of the demographic history on HLA alleles [35]. However, documented cases from modern cohorts suggest that population bottlenecks tend to affect loci unevenly [36]. The low diversity across all loci in our EHG sample is therefore unlikely to reflect an ancestral bottleneck. Second, the HLA diversity has been shown to be positively correlated with pathogen diversity in human populations [37]. Therefore, the low HLA diversity in Sakhtysh may indicate low pressure from multiple pathogen taxa on this population. However, this does not exclude frequent exposure to a few bacteria, viruses, parasites or other environmental factors that may have driven selection of certain HLA-B alleles. Characterization of the pathogen diversity in other ancient hunter-gatherer populations is therefore crucial. Furthermore, it will be important to establish whether such reduced HLA diversity is a general feature of post-glacial European hunter-gatherers.

The lower diversity in Sakhtysh is accompanied by the very high frequency of one or a few alleles for each locus. The most prominent example is B*27:05 which reached almost 50% in frequency (Additional file 2: Fig. S13). While an even distribution of allele frequencies within populations is considered a hallmark of balancing selection [38], the dominance of one allele might indicate directional selection. In line with this, HLA-B is the only locus in Sakhtysh showing a trend towards directional selection in the EW test (Additional file 1: Table S7). B*27:05 is very common both in the WHG (42%, estimation based on tag-SNP) [18, 19] and in Neolithic populations (> 20%) [17, 18]. The main ancestry component of the EF can ultimately be traced via Anatolian farmers to Anatolian hunter-gatherers [39, 40]. Although balancing selection maintains HLA alleles over deep evolutionary timescales, observations from contemporary populations indicate that, over shorter timescales, allele frequencies are shaped primarily by the demographic history and the genetic ancestry [37, 41, 42]. Therefore, despite the lack of HLA data from Anatolian hunter-gatherers, it is plausible to hypothesize that B*27:05 was already common in this group. Overall, it appears that this allele was frequent in distinct ancient populations and decreased in frequency more recently. B*27:05 shows a protective effect against chronic infections caused by hepatitis C virus and human immunodeficiency virus [43, 44], suggesting strong viral pressures on hunter-gatherers. However, it is also a strong risk allele for several inflammatory diseases such as ankylosing spondylitis and reactive arthritis that are commonly triggered by bacterial infections [45]. The decreasing frequency of B*27:05 after the Neolithic could be the result of shifting selective pressure from viral to bacterial infections [19]. This observation is also concordant with other findings suggesting an increased tolerance against intracellular pathogens since the Neolithic [46].

Another pair of alleles reaching frequencies of up to 40% in the Sakhtysh population are DRB1*08:01 and DQB1*04:02. These alleles potentially form a haplotype as they co-occur in four individuals, all of whom are homozygous in DRB1 and DQB1. Both alleles were absent in the EF and became very common in the LF. This increase has been suggested to result from the admixture of the EF with the WHG based on local ancestry inference [18]. The high frequency of this haplotype in Sakhtysh shows that it was frequent in both the EHG and WHG, possibly inherited from their common ancestral Paleolithic population 14000 years ago. A similar pattern is also observed for the DRB1*09:01-DQB1*03:03 pair (Additional file 2: Fig. S13). Despite these similarities, it can be assumed that the EHG and WHG may well have differed in parts of their HLA pools. One way to identify these differences is to look in particular at those alleles that are missing in Sakhtysh, such as DRB1*01:01 and DQB1*05:01, but appear at high frequencies in the LF. Alternatively, alleles common in Sakhtysh that are not observed in the LF (e.g., DRB1*14:01, DQB1*05:03) could be the remnants of the ANE ancestry.

The DRB1*15:01 allele was suggested to have been under selection, possibly as a result of novel pathogenic pressures accompanying changes in diet and lifestyle [18–20, 47]. The lack of DRB1*15:01 in Sakhtysh and Neolithic farmers (EF, LF) is intriguing, given its high frequency in modern Europeans. This allele was probably introduced into Europe via Steppe herders at the end of the Neolithic [18, 19]. Since the Steppe ancestry was formed by the admixture of the EHG and Caucasus hunter-gatherers (CHG) in roughly equal parts [1, 48], the lack of DRB1*15:01 in the Sakhtysh individuals suggests that this allele might have been part of the CHG HLA allele pool. However, it should be noted that because of the limited sample size (*n* = 18 for DRB1 genotyped individuals at the second-field level), we might have missed the allele in Sakhtysh if it was present at a frequency lower than 5% (one-sided binomial test, *p* > 0.05). Therefore, we cannot exclude the possibility that DRB1*15:01 introgressed from EHG into the Steppe population where it was under positive selection [20].

## Conclusions

Overall, our results underscore a high degree of genetic continuity in Sakhtysh that persisted across cultural transitions from the Lyalovo to the Late Volosovo period over two millennia. This hunter-gatherer population exhibits limited HLA diversity alongside elevated frequencies of specific alleles, a pattern that is unexpected when compared to modern outbred populations. As with all studies applying targeted HLA genotyping to aDNA, our findings should be considered in light of the inherent challenges of working with small, temporally dispersed samples. Moreover, it highlights the importance of generating additional HLA data from prehistoric hunter-gatherers and other ancient populations to contextualize and refine our understanding of past immunogenetic variation.

## Methods

### Sampling

We sampled a total of 84 skeletal elements from both petrous bones and teeth, respectively, corresponding to 43 individuals for aDNA analysis from the burial sites of Sakhtysh (Additional file 1: Table S1).

### DNA extraction and library preparation

All samples were forwarded to DNA extraction and partial uracil-DNA glycosylase library conversion [49] following strict clean-room laboratory guidelines for aDNA work [50]. Whole genome shotgun sequencing was performed on the Illumina HiSeq 6000 platform (2 × 100 bp) of the Institute of Clinical Molecular Biology in Kiel. Additionally, targeted in-solution enrichment of the HLA region was done on 31 individuals for which petrous bone was available (Additional file 1: Table S1).

### Metagenomic pathogen screening

All sequenced datasets were screened for the presence of bacterial and viral pathogens using the MEGAN Alignment Tool “MALT” v0.4.1 [51, 52], running in semi-global alignment mode and a minimum percent identity of 90% to align the samples against a database of 27,730 bacterial and 10,543 viral complete genomes [53]. Thereafter, alignments were visualized using the Metagenome Analyser “MEGAN6” v6.25.9 [51]. Positive samples were subsequently authenticated by mapping to the respective whole genome references (Additional file 1: Table S5-S6).

### Processing of reads and mapping to the human reference

Adapter sequences were removed and paired-end reads were combined using ClipAndMerge version 1.7.7 [54]. Subsequently, alignment to both the human genome (build hg19) and the human mitochondrial genome references was conducted using BWA version 0.7.15 [55], employing relaxed mapping stringency parameters (flag -n 0.01 -l 300) to accommodate anticipated mismatches in aDNA samples. Data from 18 Sakhtysh individuals previously published [1] (Additional file 1: Table S1) was downloaded. Fourteen of these individuals were sequenced separately both in this study and in [1]. Libraries from the same individuals were combined into a single BAM file with samtools v 1.12 [56] (Additional file 1: Table S1). Duplicate reads were identified and excluded using DeDup version 0.12.1 [54].

### Contamination assessment and molecular sex estimation

To evaluate the authenticity of aDNA, we examined the terminal damage of reads by quantifying the frequency of C to T substitutions using DamageProfiler version 1.1 [57]. Following validation, the initial two positions from both the 5′ and 3′-ends of the reads were trimmed using bamUtil version 1.0.15 [58]. Estimation of mitochondrial DNA contamination involved analyzing sequence deamination patterns and fragment length distributions with Schmutzi version 1.5.5.5 [59]. In male samples, contamination was additionally assessed by evaluating X-chromosome heterozygosity with ANGSD version 0.935 [60]. As these samples are precious and contamination estimates can be largely imprecise, the placement of individuals on the PCA plot as well as mitochondrial DNA/Y chromosome haplogroups were used to determine if samples should be excluded. The molecular sex was ascertained utilizing two methods that evaluate the ratio of sequences aligning to the sex chromosomes versus autosomes [61, 62].

### Estimation of mitochondrial DNA and Y-chromosome haplogroups

Mitochondrial DNA haplogroups were ascertained using HaploGrep2 [63], while Y-chromosome haplogroups were determined employing yHaplo [64]. A mapping and base quality threshold of 30 was applied.

### Estimation of biological relatedness

Kinship up to the second degree was estimated using pairwise mismatches with READv2 [65]. The first run was performed on individual libraries to confirm whether the sequence data from different sequencing runs and/or from different skeletal elements (e.g., tooth and petrous bone) were from the same individuals. Data confirmed coming from the same individuals were merged. The second run with READv2 was then performed on the merged datasets to identify kinship among Sakhtysh individuals from all phases. Since the reliability of third-degree kinship assignments using low coverage data can be compromised, leading to a higher risk of false positives [65], we limited the kinship assignments to the second degree.

### Genotyping and merging with publicly available data

We utilized SequenceTools (https://github.com/stschiff/sequenceTools) version 1.2.2 to produce pseudo-haploid genotypes at 1233013 SNP positions. Samples with fewer than 20000 genotyped SNPs were omitted from the population genetics analyses. The genotyped samples (*n* = 36) were combined with the Allen Ancient DNA Resource (AADR) reference panel (version 54.1.p1), which encompasses previously reported genotypes from more than 16000 ancient and contemporary individuals [66]. Additionally, we included samples from recent publications that were not part of the latest AADR release [1, 5, 9, 67, 68].

### Principal component analysis (PCA)

The PCA was performed on the HO panel with smartpca [67]. The calculation of principal components (PCs) was based on a subset of 64 modern populations from West Eurasia (listed in Additional file 1: Table S9) with the “lsqproject: YES” parameter. The remaining individuals from the merged dataset were projected on the calculated PCs.

### Unsupervised admixture

The merged HO genotype data was pruned in PLINK v1.90b6.21 [69], with an r^2^ threshold of 0.4, a window size of 200 and a step size of 25 (command “–indep-pairwise 200 25 0.4”) prior to running the unsupervised admixture analysis with the software ADMIXTURE v1.3.0 [70]. We then ran ADMIXTURE with 3 to 12 components (K) and 100 bootstraps each. Cross-validation error was calculated for each model to identify the best K component by using the flag “–cv”.

### F-statistics

Shared genetic drift between Sakhtysh and published ancient groups was calculated with the program qp3Pop [71] in the format f_3_(Sakhtysh, test; Mbuti), where “test” refers to published populations available in the 1240K dataset dating between 4000 and 9000 cal BP. F_4_ statistics in the format f_4_(Mbuti, test; Sidelkino, Villabruna HG) and f_4_(Mbuti, test; AfontovaGora3, Villabruna HG) was calculated with qpDstat [71] with the parameter “f4mode: YES". The “test” in the f_4_ tests refers to Sakhtysh and other selected ancient populations from the 1240K dataset. Two-way admixture models were tested with qpAdm [71] using a set of outgroups listed in Additional file 1: Table S2. The parameter “allsnps: YES” was applied for qpAdm modeling.

### Testing genetic continuity

We used f_3_ statistics to investigate the level of genetic continuity among individuals of Sakhtysh throughout the different periods with the test f_3_(IIa grave 40, test; Mbuti) [9]. “IIa grave 40” is the second highest covered individual and also the second oldest sample in the dataset (Additional file 1: Table S1), and “test” is the other individuals from the site. With these f_3_ statistics, we defined genetic continuity as the absence of substantial genetic shifts between individuals from different periods (i.e. all individuals share a high and statistically indistinguishable level of genetic drift with the reference individual from the site, regardless of the burial date). Under continuity, genetic similarity should remain high across the ~ 2000-year sampling span, whereas a violation of continuity would manifest as significantly lower f_3_ values for some temporal groups, indicating that they share less drift with the reference individual than others. In addition to the f_3_ statistics, we implemented an approach using pairwise f_4_ statistics in the format f_4_(Mbuti, sampleA; sampleB, outgroup). Under a model of genetic continuity, these f_4_ values should cluster within the same range regardless of the temporal distance between sample pairs, as both samples would represent the same underlying population with no differential genetic drift relative to the outgroup since their shared divergence from Mbuti. We tested multiple outgroup populations that are genetically closer to our Sakhtysh samples than the deeply divergent Mbuti (shown in Additional file 2: Fig. S7) to ensure our results are robust across different reference populations. For both f-statistics, we only present results from tests with at least 10000 SNPs.

### HLA analysis

Accurate HLA genotyping in ancient samples requires targeted enrichment of HLA sequences rather than genome-wide SNP data. The 1240K SNP panel used for genome-wide analyses in this study is unsuitable for assessing HLA diversity. This panel is highly enriched in common variants to the extent that the expected diversity difference between the major histocompatibility complex (MHC) and the rest of chromosome 6 is reversed (Additional file 2: Fig. S15). This bias, compounded by the use of pseudohaploid genotype calls in low-coverage ancient samples and known reference mapping bias at HLA loci [72], produces misleading SNP-based HLA diversity estimates for ancient samples. This observation underscores why targeted capture-based HLA genotyping methods are necessary and superior for this region. Therefore, genotyping of the enriched HLA sequence data was performed using the TARGT pipeline [23]. HLA class-I calls were also confirmed using OptiType [24] and only concordant genotype calls were used for analysis. All analyses were performed both at the first-field and the second-field resolution. Population samples for comparative analysis include two ancient datasets of central European Neolithic farmers, five modern European populations from the 1000 Genomes Project and one modern Russian population (labeled MR) from the Allele Frequency Net Database (Additional file 1: Table S8) [18, 25, 26]. HLA allele frequencies were calculated within each population based on the genotypes by direct counting of alleles. The exact test for HWE and the EW test of neutrality were performed using Pypop [73, 74]. HLA diversity was quantified using Shannon’s diversity index (H’) [75]. In order to account for the small size of the Sakhtysh sample compared to the rest of the population samples, 100 random sub-samples of each population were generated based on the allele frequencies and with size equal to the Sakhtysh sample. HLA diversity of populations was compared based on the distribution of Shannon’s diversity index of these sub-samples. Pairwise F_ST_ values were calculated by using the *hierfstat* package [75, 76].

## Supplementary Information

Additional file 1: Table S1. Sample information and summary of population genetic results. Table S2. qpAdm modeling. Table S3. Kinship analysis. Table S4. Bacteria detected during pathogen screening. Table S5. Bacteria references used for mapping. Table S6. HBV references used for mapping. Table S7. Summary of Hardy–Weinberg and Ewens-Watterson neutrality tests. Table S8. Modern populations used for the comparative HLA analysis. Table S9. List of population used for PCA

Additional file 2: Figure S1. Outgroup f_3_ statistics. Figure S2. Unsupervised admixture. Figure S3. f_4_ statistics of Sakhtysh allele sharing relative to EHG and WHG. Figure S4. f_4_ statistics of Sakhtysh allele sharing relative to ANE and WHG. Figure S5. Genetic continuity f_3_ test. Figure S6. Genetic continuity assessment using pairwise f_4_ statistics. Figure S7. Correlation between genetic distances and radiocarbon dates. Figure S8. Results of kinship analysis. Figure S9. Damage plots. Figure S10. HLA diversity measured at first-field resolution. Figure S11. HLA diversity measured at second-field resolution. Figure S12. Frequencies of HLA alleles at first-field resolution. Figure S13. Frequencies of HLA alleles at second-field resolution. Figure S14. PCoA plots based on pairwise FST values calculated using second-field HLA allele frequencies. Figure S15. Expected heterozygosity within the MHC region and the rest of chromosome 6.

## Data Availability

Raw sequence data reported in this study are available from the European Nucleotide Archive (ENA) with the accession code PRJEB81456 [77]. Previously published genotype data for ancient and present-day individuals used in the population genetic analyses were obtained from the Allen Ancient DNA Resource (AADR) v54.1.p1, available at https://dataverse.harvard.edu/dataset.xhtml?persistentId=doi:10.7910/DVN/FFIDCW. The corresponding original publications are listed in the AADR documentation or cited in the manuscript [1, 5, 9, 66–68]. HLA genotype data for modern individuals were obtained from the 1000 Genomes Project, available at http://ftp.1000genomes.ebi.ac.uk/vol1/ftp/data_collections/HLA_types/ and the Allele Frequency Net Database (https://www.allelefrequencies.net/pop6001c.asp?pop_id=3744) [25, 26]. Custom R scripts used for the analysis of the HLA data are available on GitHub at https://github.com/onur-ozer/EHG-immunogenomics with the source code is released under the MIT license. The version of the code used in this manuscript is deposited in Zenodo (DOI: 10.5281/zenodo.21197801) [78].
