# Supplementary material for: Ancient genomics study reveals low HLA diversity in eastern hunter-gatherers
Source: Genome Biol. 2026 Jul 29;27:243. doi: 10.1186/s13059-026-04214-8 (PMC13418142; doi:10.1186/s13059-026-04214-8)
Supplement: Supplementary file 2 — Additional file 2: Figure S1. Outgroup f3 statistics. Figure S2. Unsupervised admixture. Figure S3. f4 statistics of Sakhtysh allele sharing relative to EHG and WHG. Figure S4. f4 statistics of Sakhtysh allele sharing relative to ANE and WHG. Figure S5. Genetic continuity f3 test. Figure S6. Genetic continuity assessment using pairwise f4 statistics. Figure S7. Correlation between genetic distances and radiocarbon dates. Figure S8. Results of kinship analysis. Figure S9. Damage plots. Figure S10. HLA diversity measured at first-field resolution. Figure S11. HLA diversity measured at second-field resolution. Figure S12. Frequencies of HLA alleles at first-field resolution. Figure S13. Frequencies of HLA alleles at second-field resolution. Figure S14. PCoA plots based on pairwise FST values calculated using second-field HLA allele frequencies. Figure S15. Expected heterozygosity within the MHC region and the rest of chromosome 6. [file 13059_2026_4214_MOESM2_ESM.pdf]

Supplementary Material for

Ancient genomics study reveals low HLA diversity in Eastern Hunter-Gatherers

Authors

Onur Özer[1], Nicolas Antonio da Silva[1], Magdalena Haller-Caskie[1], Daniel Anton Myburgh[1], Henny Piezonka[2], Anastasia Khramtsova[3], Elena L. Kostyleva[4], Maria V. Dobrovol'skaya[5], Sergei V. Vasilyev[6], Elizaveta Veselovskaya[6], John Meadows[7,8], Almut Nebel[1], Ben Krause-Kyora[1]

Affiliations

[1] Institute of Clinical Molecular Biology, Kiel University, 24105 Kiel, Germany

[2] Institute of Prehistoric Archaeology, Free University of Berlin, 14195 Berlin, Germany

[3] Institute of Pre- and Protohistoric Archaeology, Kiel University, 24118 Kiel, Germany

[4] Ivanovo State University, 153025 Ivanovo, Russia

[5] Institute of Archaeology of the Russian Academy of Sciences, 117292 Moscow, Russia

[6] Institute of Ethnology and Anthropology of the Russian Academy of Sciences, Center of Physical Anthropology, 117312 Moscow, Russia

[7] Leibniz-Laboratory for AMS Dating and Stable Isotope Research, Kiel University, 24118 Kiel, Germany

[8] Leibniz-Zentrum für Archäologie, Schloss Gottorf, 24837 Schleswig, Germany

*** corresponding author**: b.krause-kyora@ikmb.uni-kiel.de

Content

Figures S1 – S15

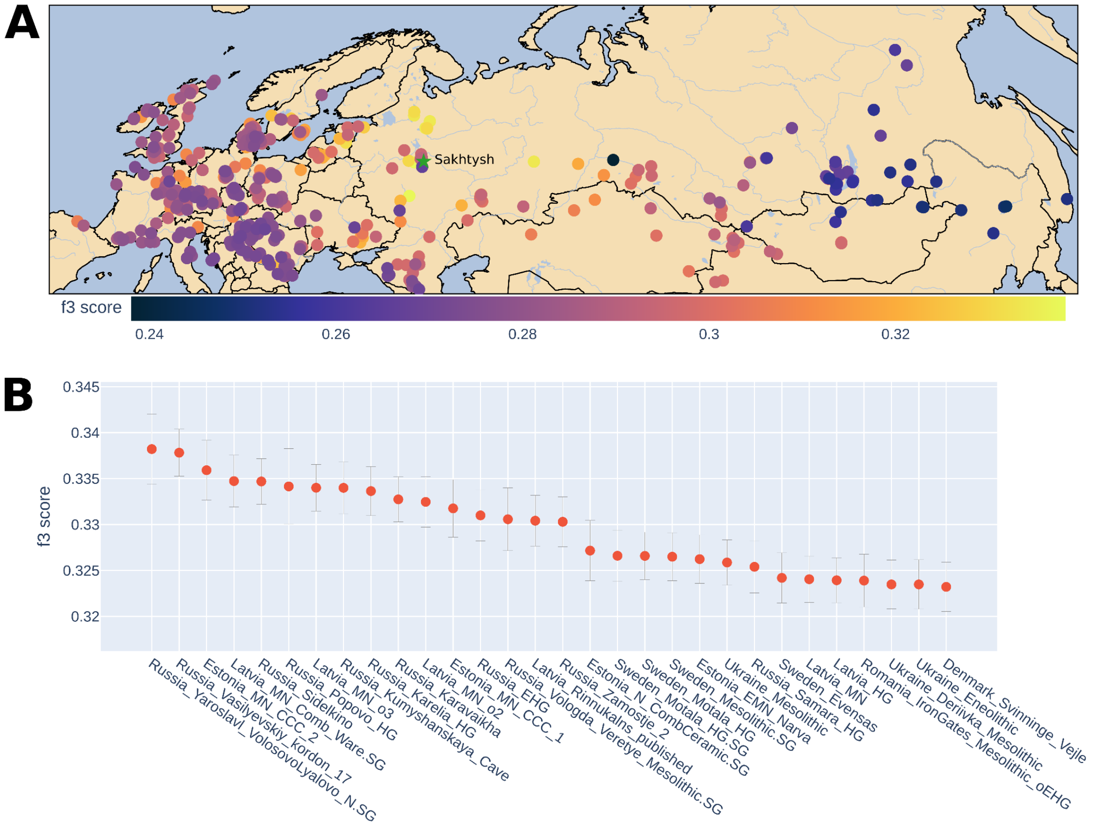

Fig. S1. Shared genetic drift among Sakhtysh and published ancient populations. The outgroup f_3 statistics was computed in the format f_3(Sakhtysh, X; Mbuti) where X is a published ancient population. **A**. Geographic distribution of the X populations tested and their respective f_3 score. **B.** The top 30 published populations with the highest f_3 score. Sakhtysh is highlighted in panel A as a green star.

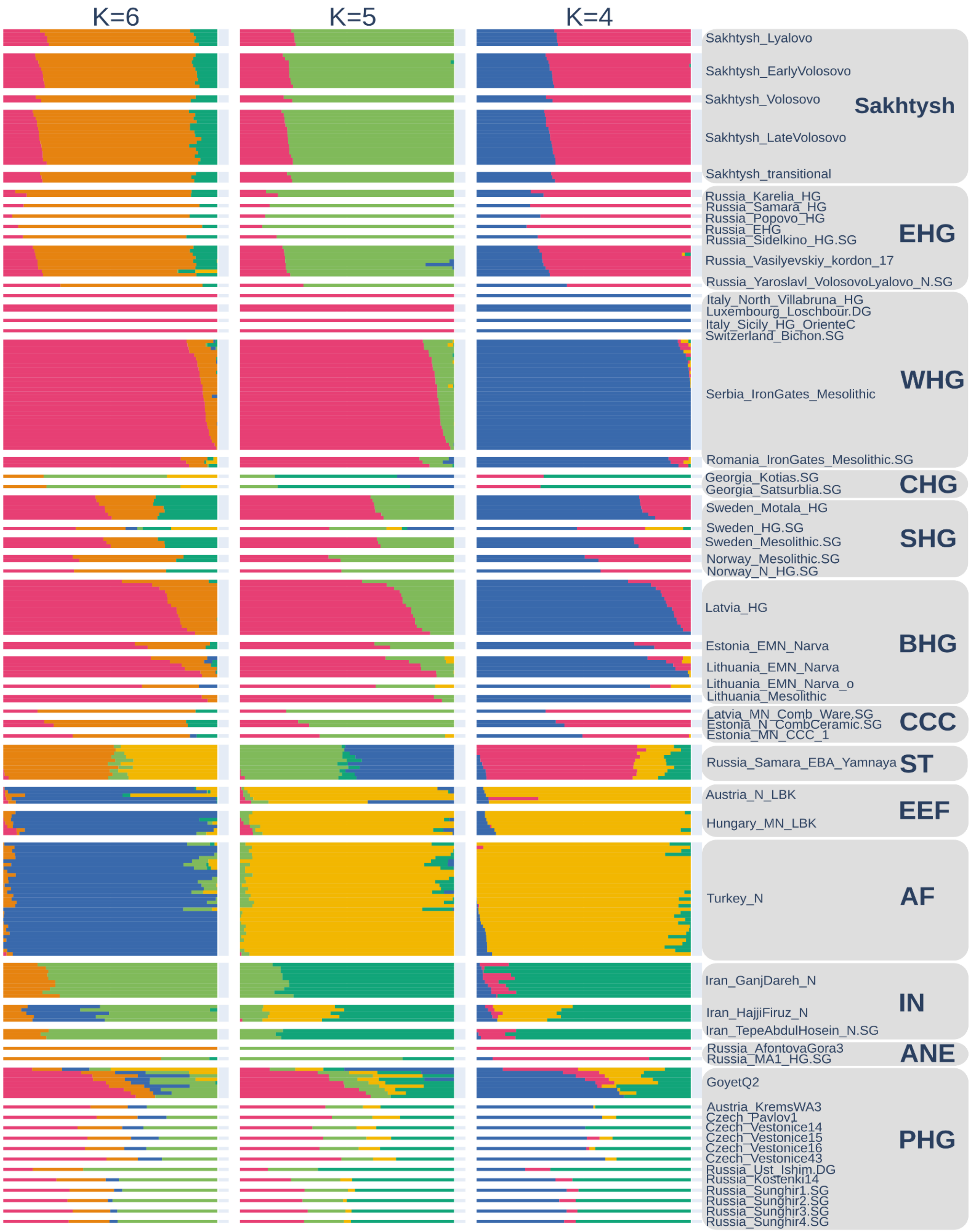

Fig. S2. Estimated ancestry proportions for the ancient individuals. The unsupervised admixture modeling was performed using 2 to 12 components (K), but only the three K with lowest cross-validation error are displayed. EHG = Eastern Hunter-Gatherers; WHG = Western Hunter-Gatherers; CHG = Caucasus Hunter-Gatherers; SHG = Scandinavian Hunter-Gatherers; BHG = Baltic Hunter-Gatherers; CCC = Comb Ceramic Culture; ST =

Steppe herders; EEF = Early European Farmers; AF = Anatolian Farmers; IN = Iran Neolithic; ANE = Ancient North Eurasian; PHG = Paleolithic Hunter-Gatherers.

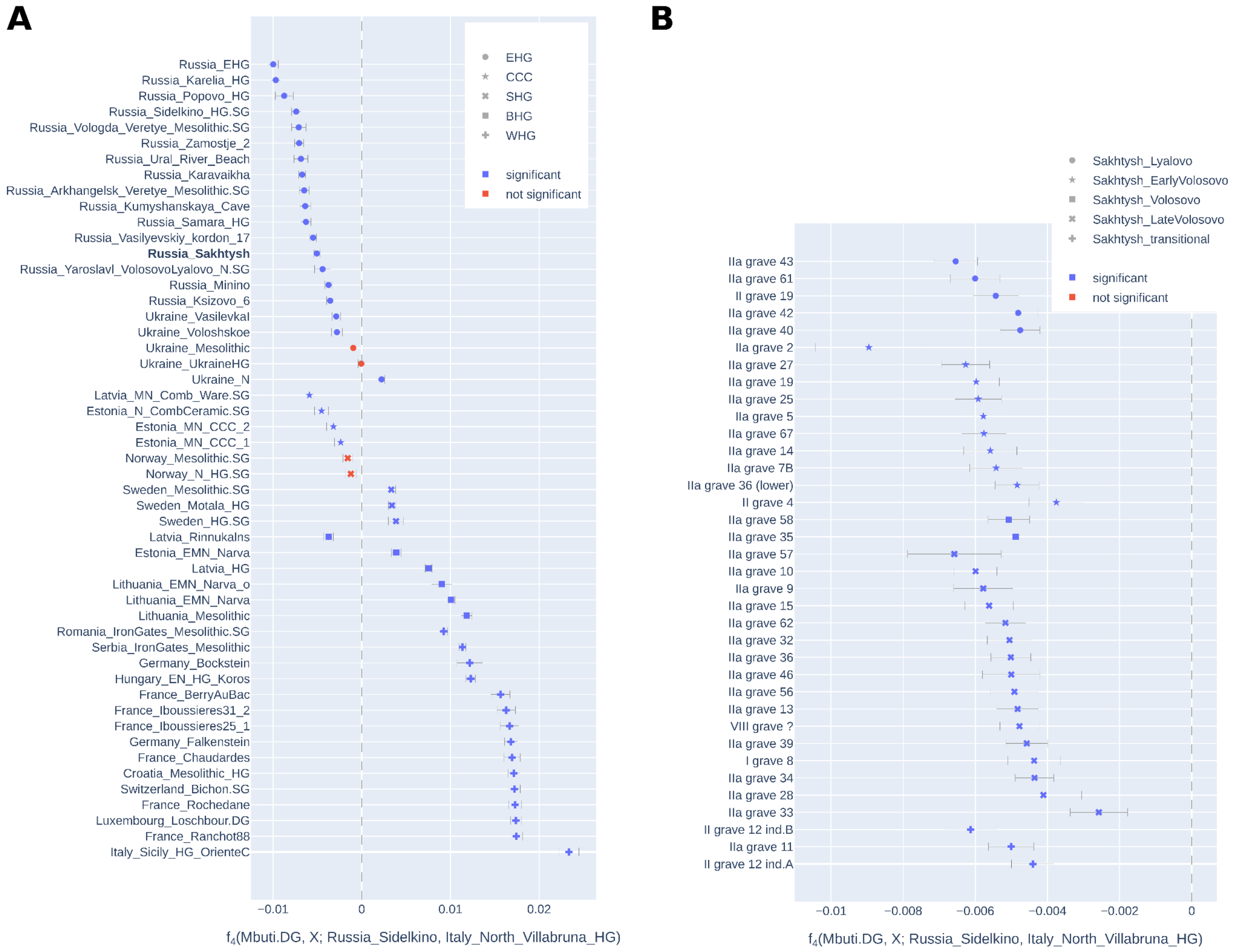

Fig. S3. A. f_4 statistics to investigate the allele sharing pattern of Sakhtysh and selected published ancient populations across Eurasia relative to EHG (Sidelkino HG) and WHG (Villabruna HG) sources. **B.** The same test was conducted on an individual level across the Sakhtysh samples dating to the different cultural periods.

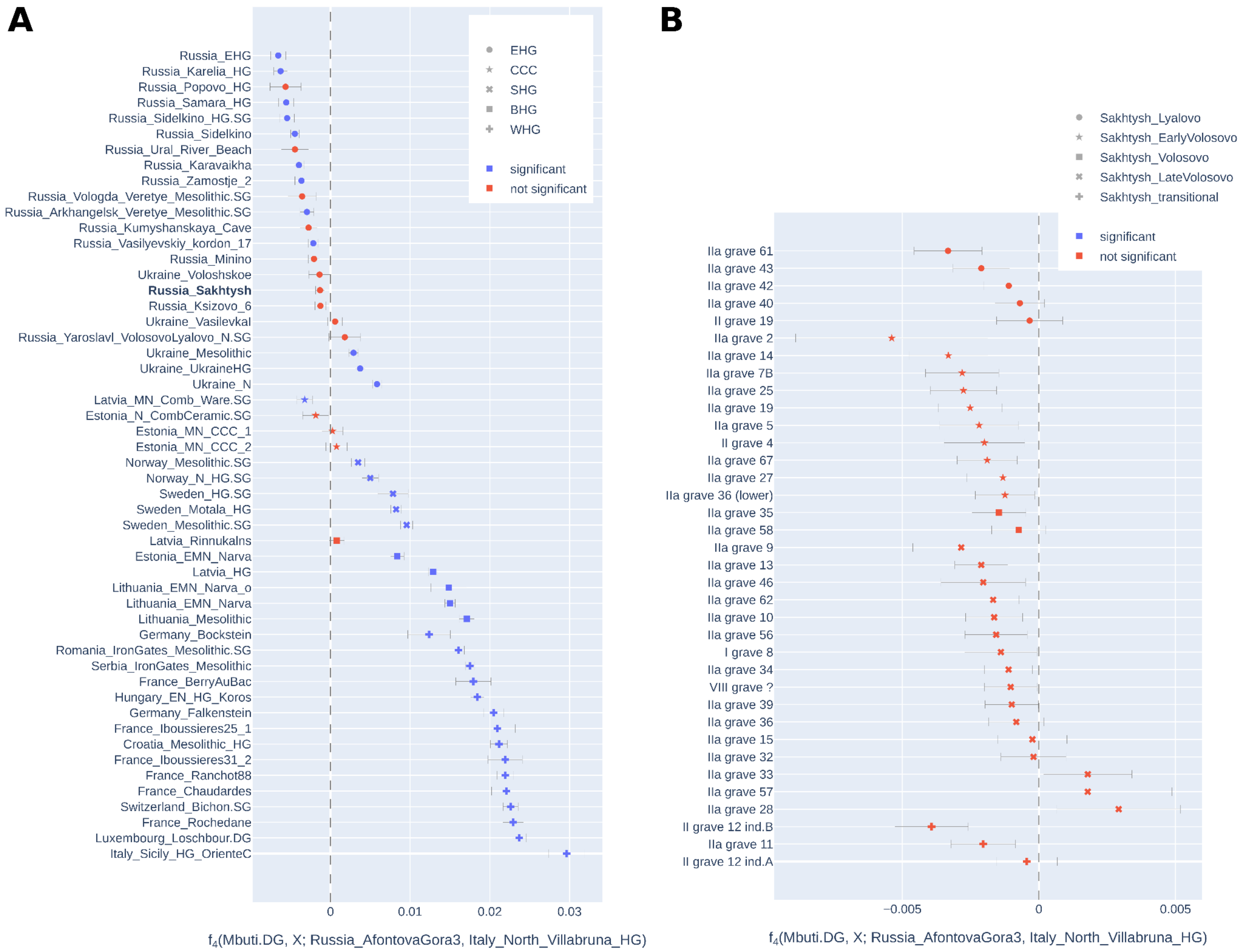

Fig. S4. A. f_4 statistics to investigate the allele sharing pattern of Sakhtysh and selected published ancient populations across Eurasia relative to ANE (AfontovaGora3) and WHG (Villabruna HG) sources. **B.** The same test was conducted on an individual level across the Sakhtysh samples dating to the different cultural periods.

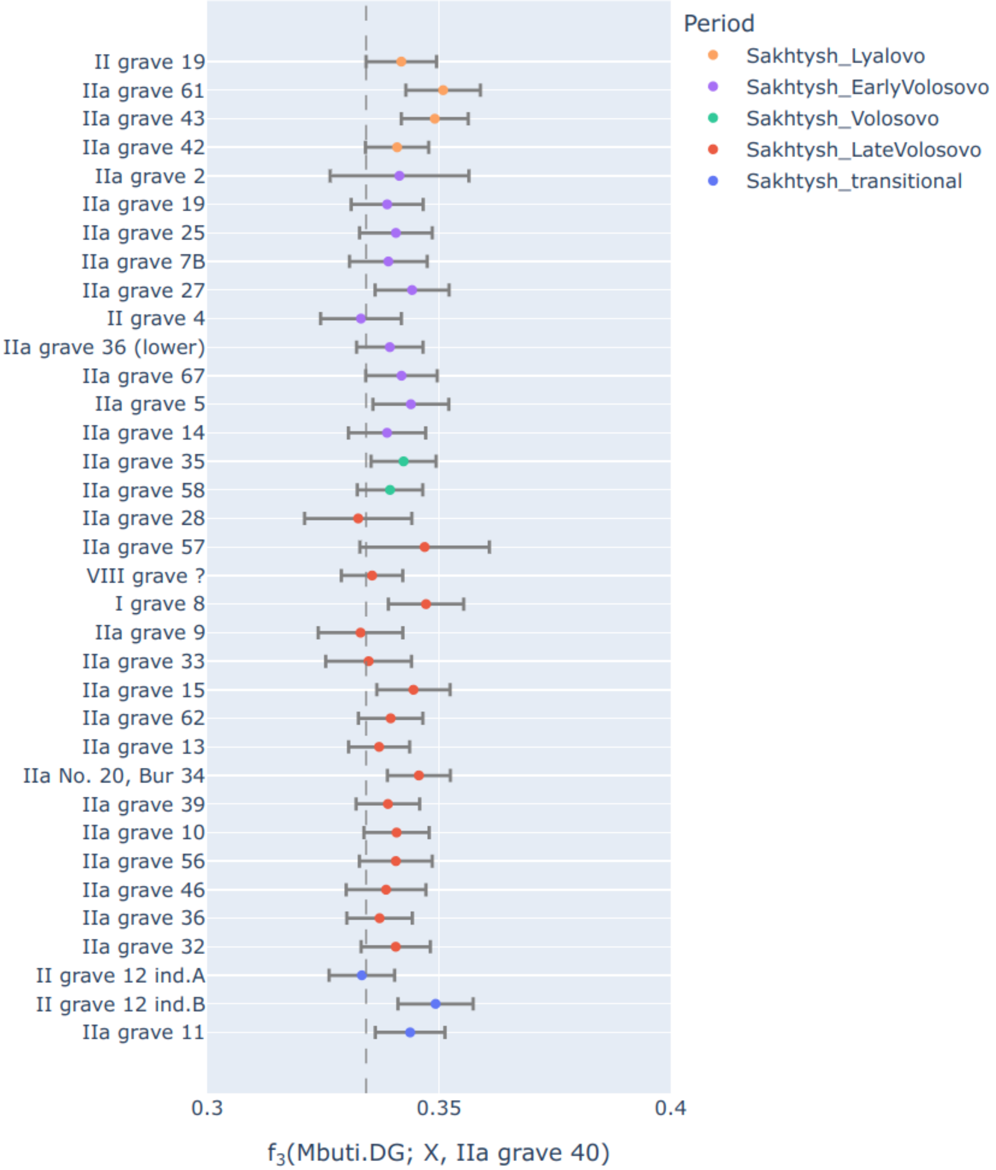

Fig. S5. Genetic continuity f_3 test across cultural periods of Sakhtysh individuals. The test, conducted in the format f_3(IIa grave 40, X; Mbuti), compares the genetic affinity of test individuals (X) with the sample having the second-highest genome coverage and the second oldest date from Sakhtysh (IIa grave 40). Individuals are ordered by assigned cultural period and median cal BP. Error bars represent 95% confidence intervals derived from block Jackknife standard errors, with the vertical line denoting the lower limit of the 95% confidence interval for comparison with the oldest dated individual.

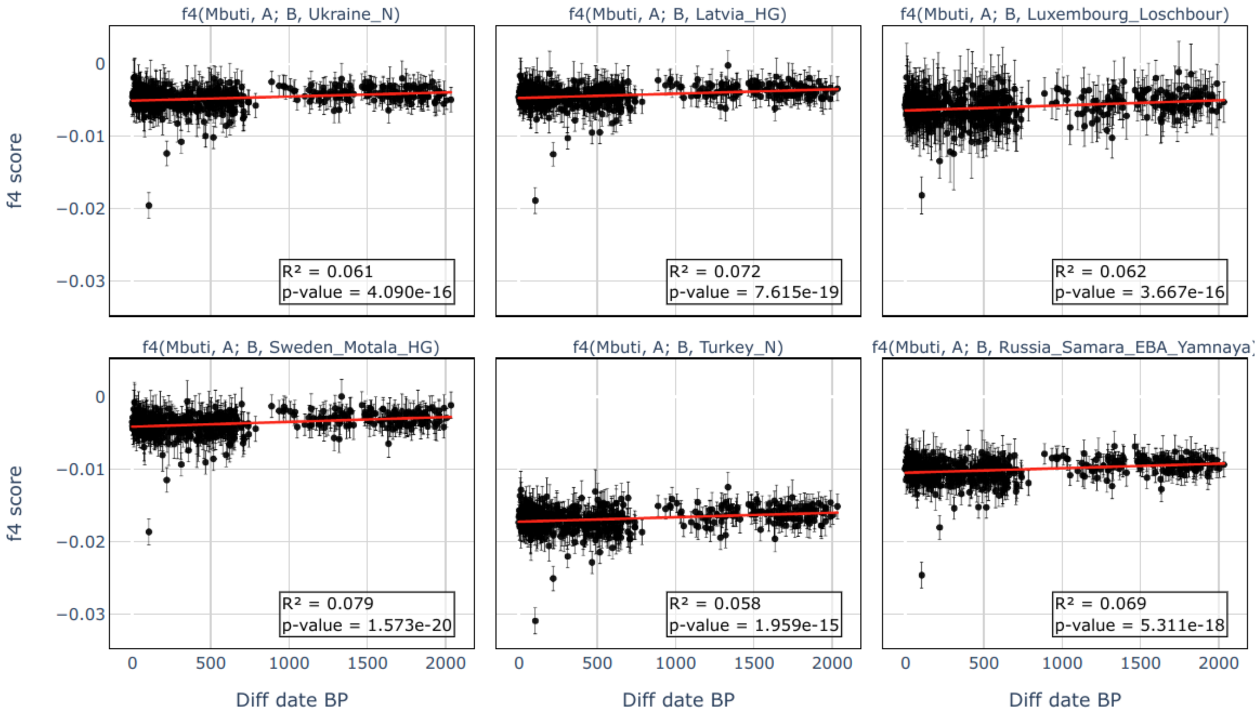

Fig. S6. Genetic continuity assessment using pairwise f_4 statistics. Panels show f_4(Mbuti, sampleA; sampleB, outgroup) tests across different outgroup populations (panel labels). Each point represents a pairwise comparison between two Sakhtysh individuals, with the x-axis showing the absolute temporal difference (years BP) between their radiocarbon dates and the y-axis showing the f_4 statistic value. outliers in the plot with very low f_4 values are related individuals (e.g.: the pair with the lowest f_4 values across all panels are individuals IIa grave 32 and IIa grave 10 reported in Sup. Table 3 and Fig S6), whose shared recent ancestry creates systematic f_4 deviations from the unrelated population baseline.

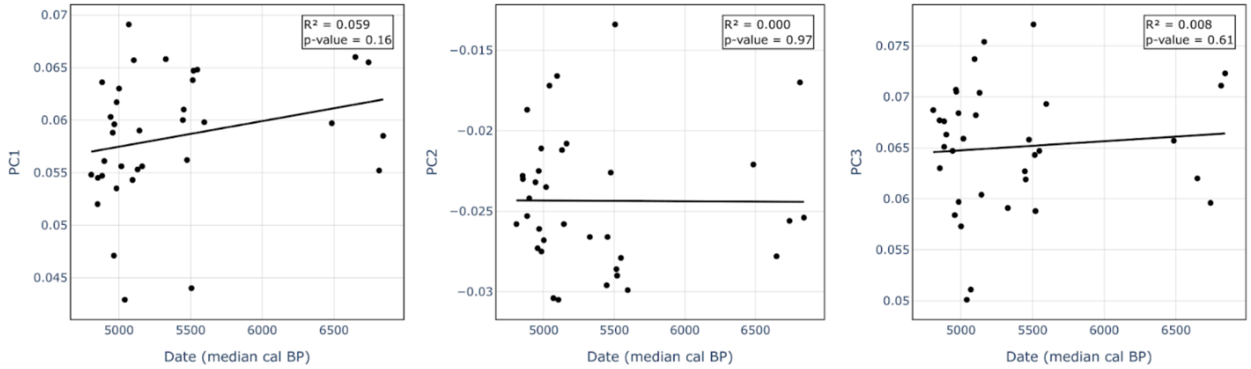

Fig. S7. Correlation between genetic distances, represented by principal component scores from PCA, and the mean date for each sample.

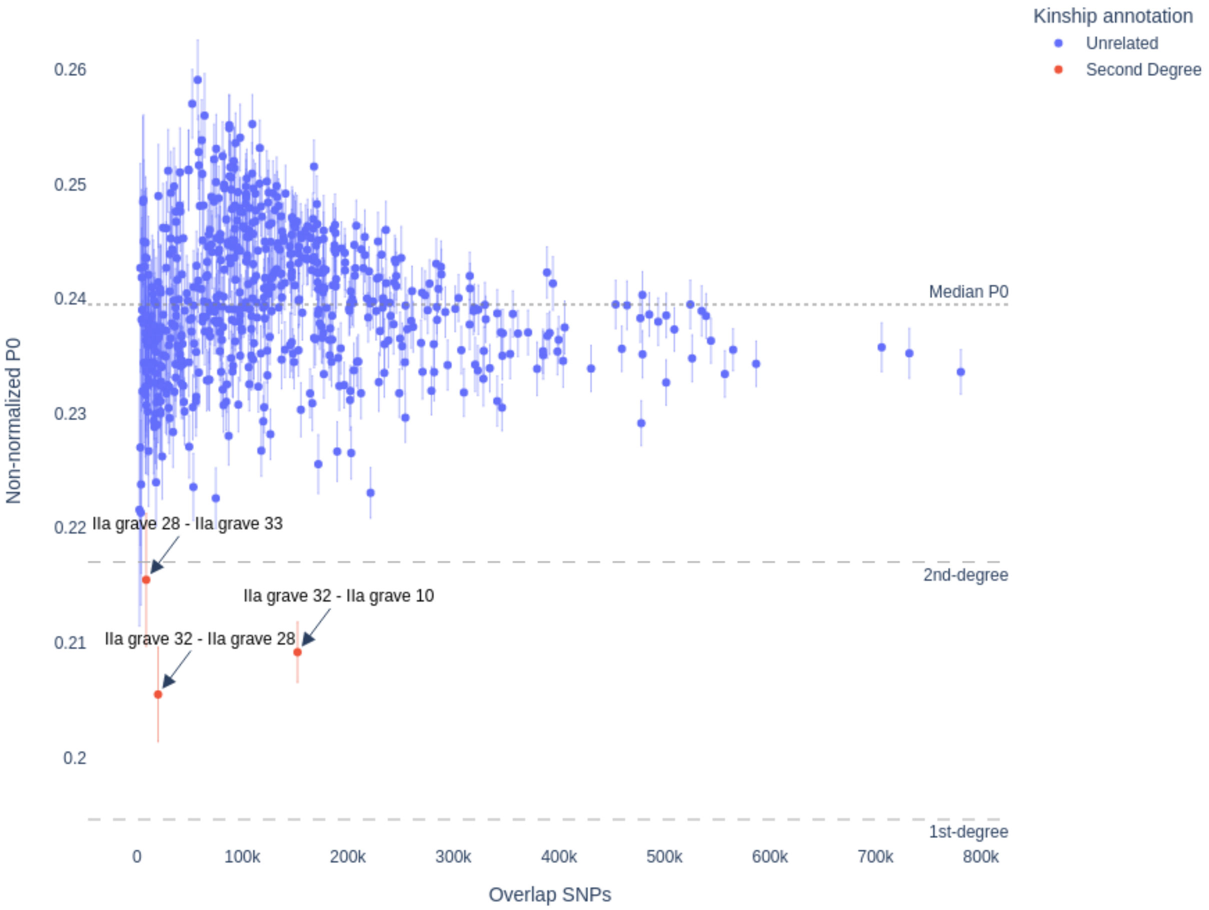

Fig. S8. Results of kinship analysis. The figure displays the non-normalized pairwise mismatch rate (P0) along the y-axis. Each symbol represents one pairwise comparison and the x-axis shows the number of overlapping SNPs between each pair. The arrows point to the comparisons that correspond to the observed 2nd degree relationships described in the Discussion.

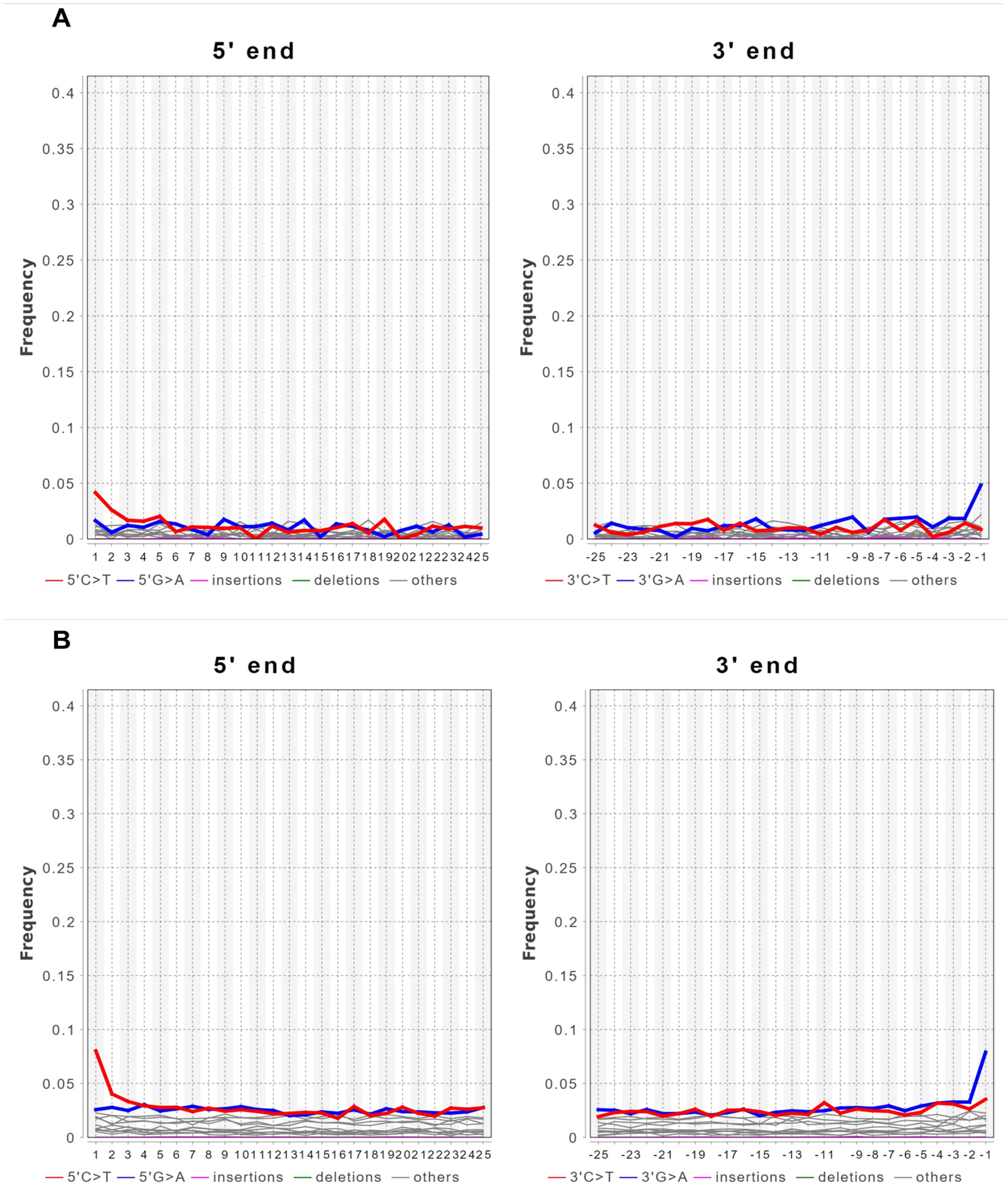

Fig. S9. DNA deamination patterns of sequenced reads mapped to the reference genome of *Erysipelothrix rhusiopathiae* strain Fujisawa (NC_015601.1). **A.** Reads generated from a tooth sample from IIa grave 65 (KH180478). The number of reads used to determine the frequencies was 2503, which represents 97.28% of all input reads. **B.** Reads generated from a tooth sample from IIa grave 58 (KH180487). The number of reads used to determine the frequencies was 14708, which represents 90.21% of all input reads.

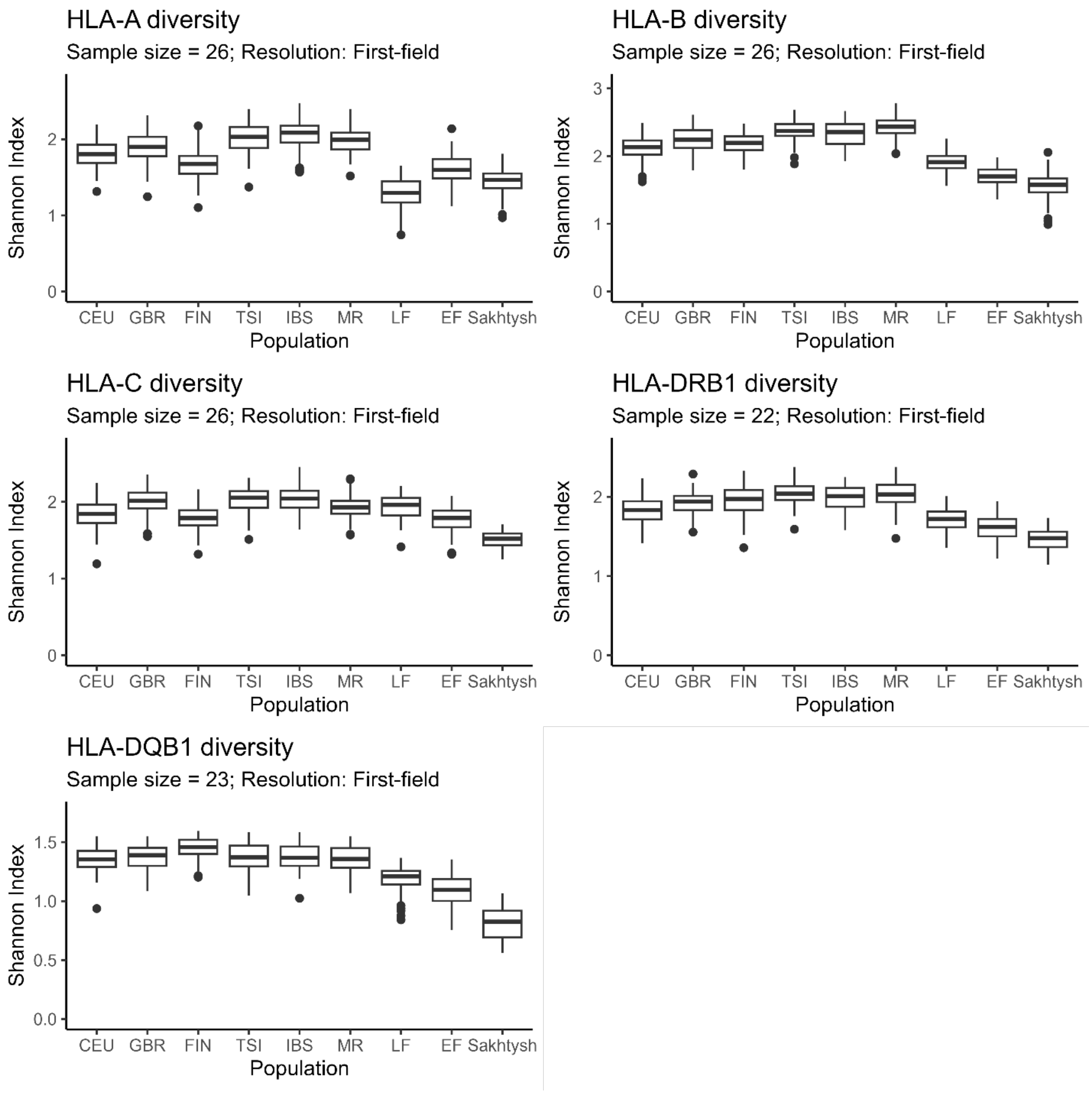

Fig. S10. HLA diversity measured at first-field resolution using the Shannon index. Boxplots represent the distribution of the H' values of 100 samples taken from each population with sample sizes indicated below headers.

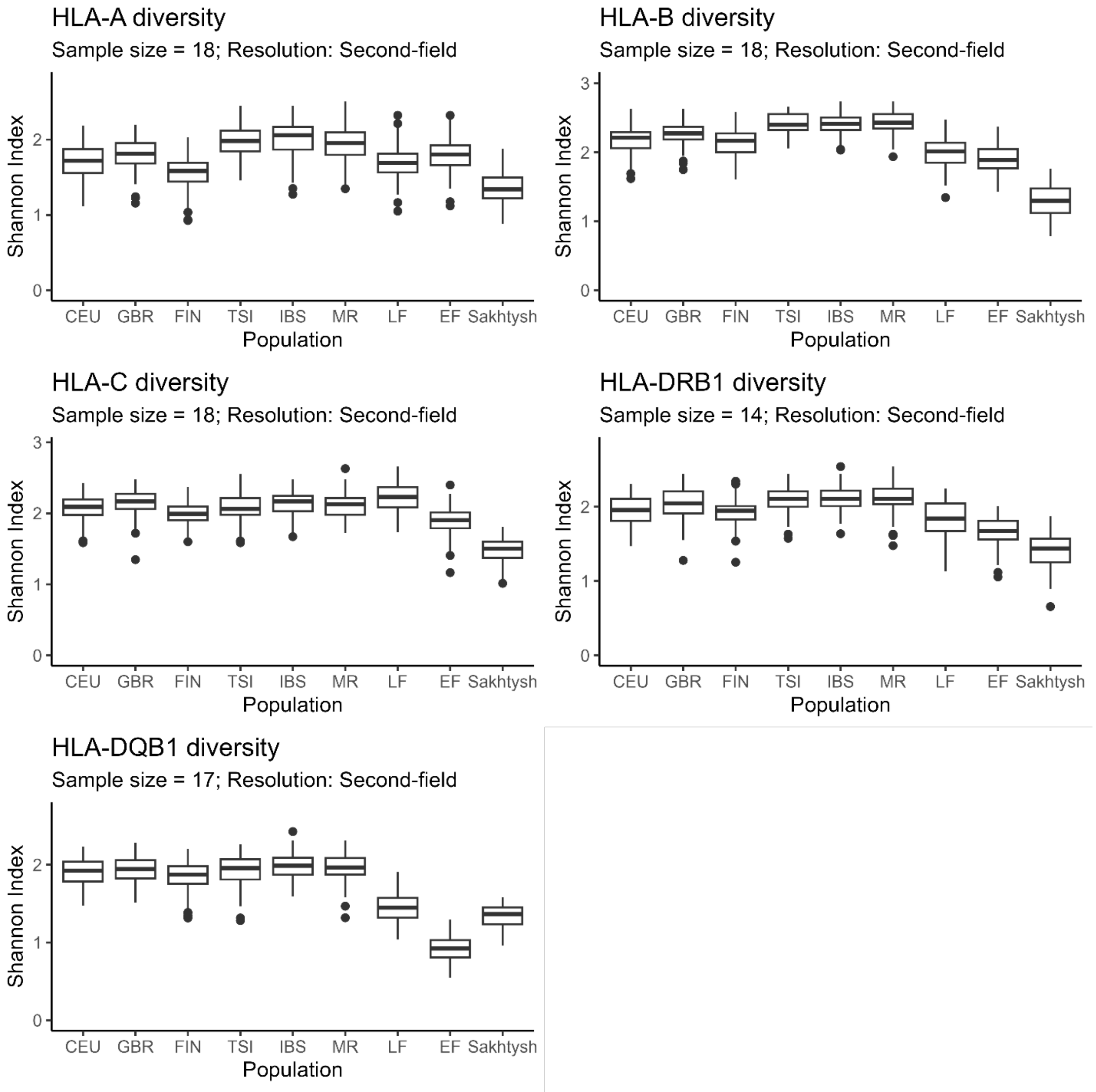

Fig. S11. HLA diversity measured at the second-field resolution using the Shannon index. Boxplots represent the distribution of the H’ values of 100 samples taken from each population with sample sizes indicated below headers.

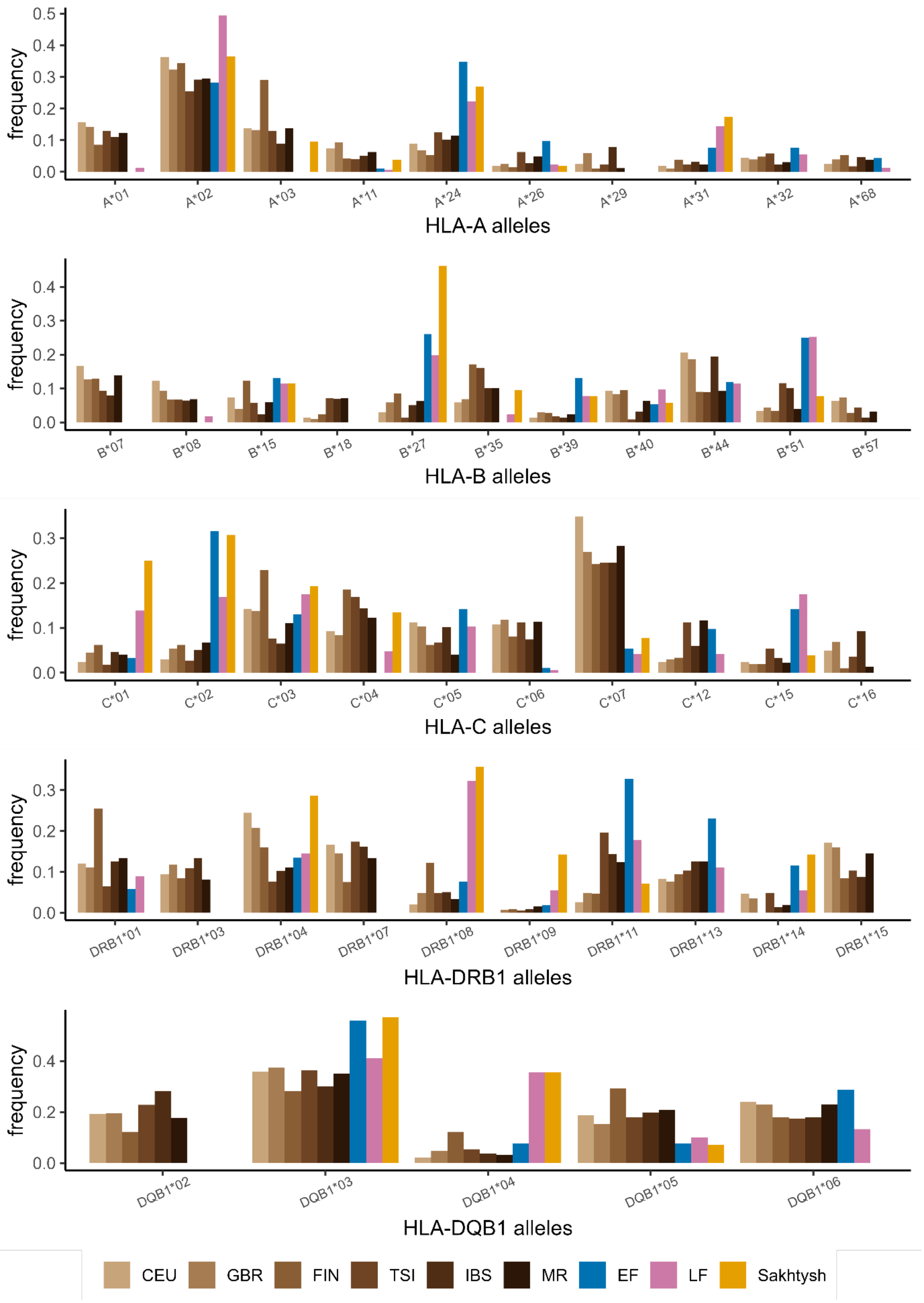

Fig. S12. Frequencies of the common alleles at the first-field resolution in each sample. Only the most common five alleles in each population were plotted.

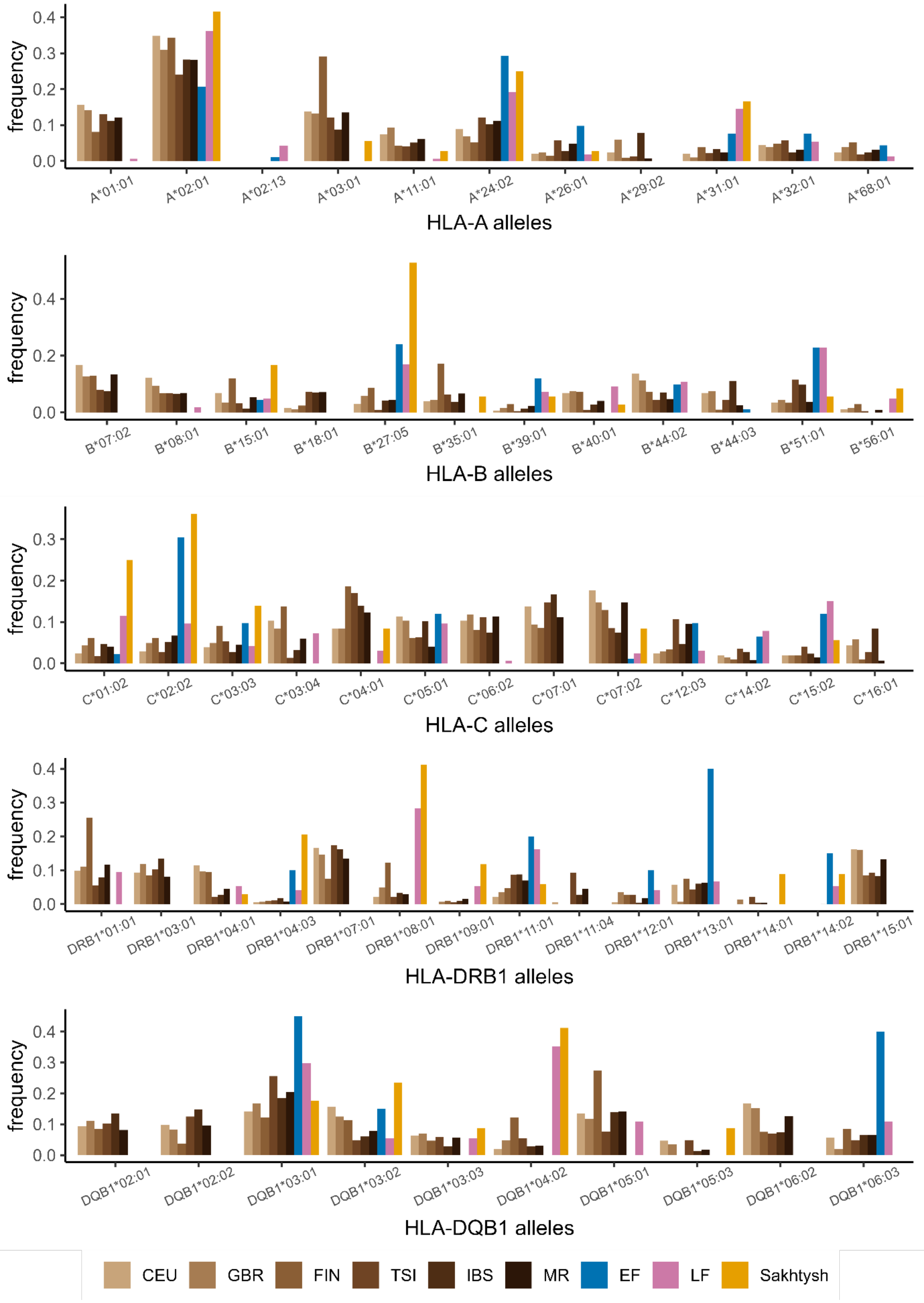

Fig. S13. Frequencies of the common alleles at the second-field resolution in each sample. Only the most common five alleles in each population were plotted.

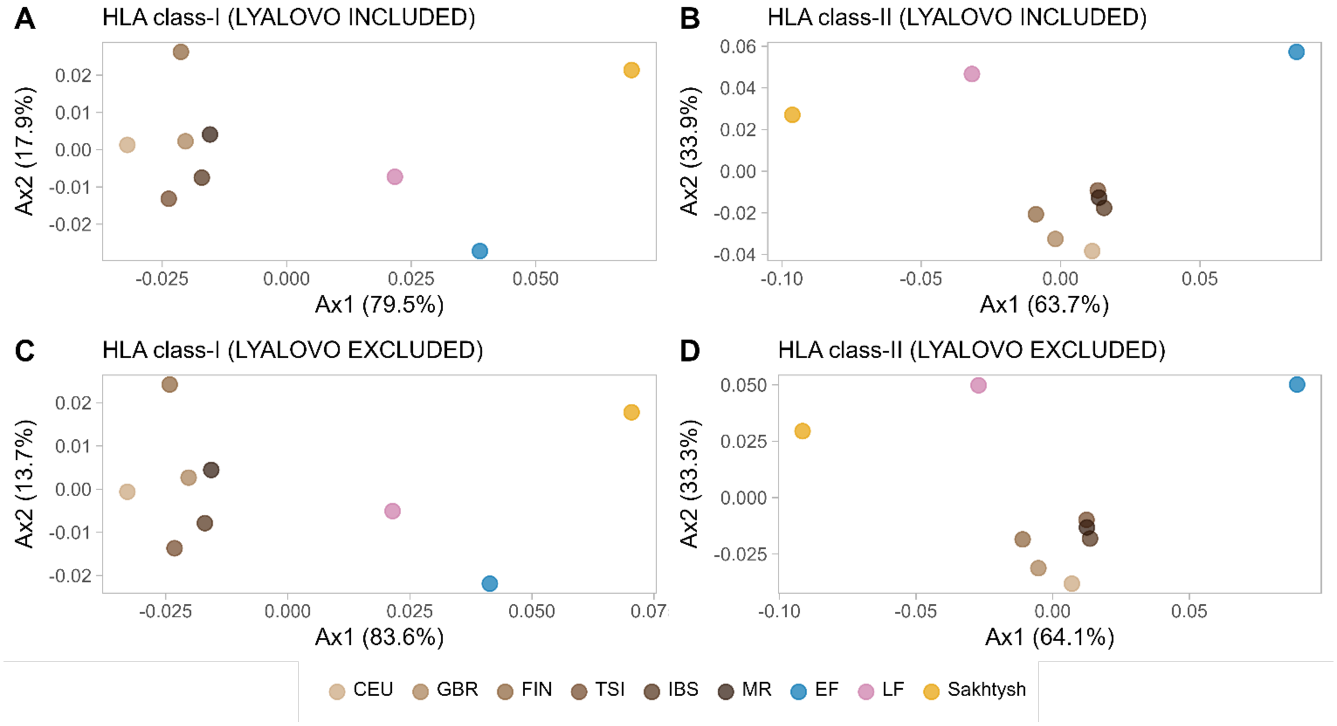

Fig. S14. PCoA plots based on pairwise F_{st} values calculated using second-field allele frequencies in order to investigate the effect of pooling all samples across cultural periods. The left column (same as Fig. 2 C & D) includes four individuals from the oldest Lyalovo period in Sakhtysh, while the right column excludes those individuals.

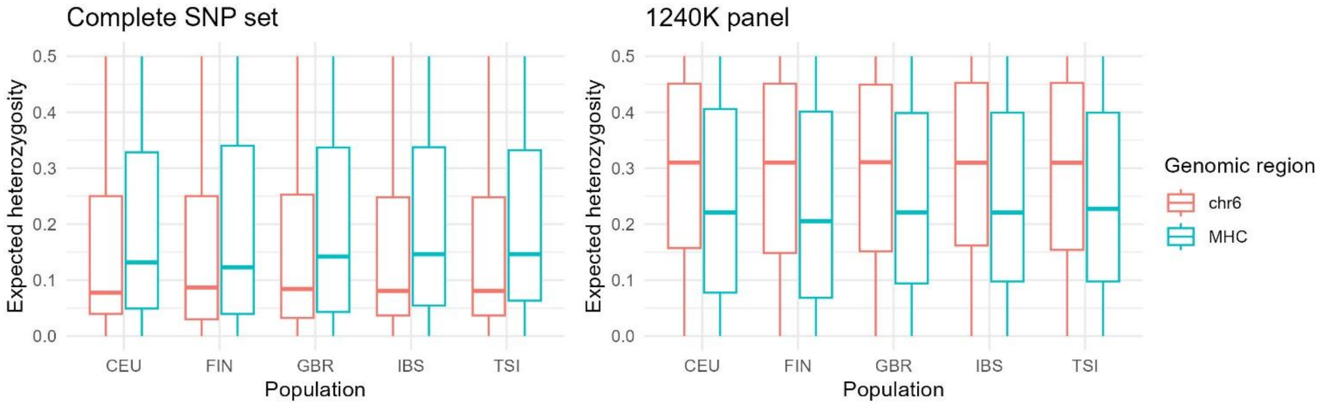

Fig. S15. Expected heterozygosity within the MHC region and the rest of the chromosome 6 for five European populations of the 1000 Genomes Phase 3 dataset (CEU, FIN, GBR, IBS, TSI). In order to assess the suitability of the 1240K SNP panel for measuring MHC diversity, we restricted the analysis to chromosome 6 and performed two parallel analyses: one using the complete SNP set from the 1000G phase 3 dataset (left panel) and one using only SNPs overlapping with the 1240K panel (right panel). In both analysis, SNPs with more than 2% missing genotypes and individuals with more than 2% missing data were removed. Variants with a minor allele frequency below 1% were filtered out. Linkage disequilibrium pruning was performed using PLINK v1.9 (--indep-pairwise 50 10 0.5). The remaining SNPs were then partitioned into the MHC region (chr6: 28,477,797–33,448,354) and the rest of chromosome 6 (labeled simply as chr6). Per-population allele frequencies were computed and expected heterozygosity (2pq) was derived for each SNP based on MAF.
